# Cefotaxime/sulbactam plus gentamicin as a potential carbapenem- and amikacin-sparing first-line combination for neonatal sepsis in high ESBL prevalence settings

**DOI:** 10.1093/jac/dkad177

**Published:** 2023-06-07

**Authors:** J B Readman, M Acman, A Hamawandi, Cheng-Hsun Chiu, M Sharland, J A Lindsay, J F Standing

**Affiliations:** Institute of Infection and Immunity, St George’s, University of London, London, UK; Infection, Immunity and Inflammation, UCL Great Ormond Street Institute of Child Health, London, UK; UCL Genetics Institute, University College London, London, UK; Institute of Infection and Immunity, St George’s, University of London, London, UK; Chang Gung Memorial Hospital, Chang Gung University, Taoyuan, Taiwan; Institute of Infection and Immunity, St George’s, University of London, London, UK; Institute of Infection and Immunity, St George’s, University of London, London, UK; Institute of Infection and Immunity, St George’s, University of London, London, UK; Infection, Immunity and Inflammation, UCL Great Ormond Street Institute of Child Health, London, UK; Department of Pharmacy, Great Ormond Street Hospital for Children, London, UK

## Abstract

**Background:**

Infection with ESBL-producing Enterobacteriaceae infection is ubiquitous in some neonatal ICUs and increasing levels of antibiotic resistance are a cause for urgent concern. Delineation of bacterial and viral sepsis can be challenging, often leading to patients receiving empirical antibiotics without or whilst waiting for a definitive causal diagnosis. Empirical therapy is often dependent on broad-spectrum ‘Watch’ antibiotics, contributing to further resistance.

**Methods:**

ESBL-producing Enterobacteriaceae clinical isolates found to have caused neonatal sepsis and meningitis underwent a detailed *in vitro* screening including susceptibility testing, chequerboard combination analysis and hollow-fibre infection model dynamic analyses using combinations of cefotaxime, ampicillin and gentamicin in combination with β-lactamase inhibitors.

**Results:**

Additivity or synergy was found for all antibiotic combinations against seven *Escherichia coli* and three *Klebsiella pneumoniae* clinical isolates. Cefotaxime or ampicillin plus sulbactam combined with gentamicin was able to consistently inhibit the growth of ESBL-producing isolates at typical neonatal doses, and the combination cleared the hollow-fibre infection model system of organisms resistant to each agent alone. The combination of cefotaxime/sulbactam and gentamicin was consistently bactericidal at clinically achievable concentrations (*C*_max_ of 180, 60 and 20 mg/L for cefotaxime, sulbactam and gentamicin, respectively).

**Conclusions:**

The addition of sulbactam to cefotaxime or ampicillin to the typical first-line empirical therapy could obviate the need for carbapenems and amikacin in settings with high ESBL-infection prevalence.

## Introduction

Infectious diseases are the leading cause of death in children under 5 years of age with bacterial infections; in particular, MDR Gram-negative organisms are of increasing concern.^1^ For example, an analysis of the change in antimicrobial resistance profiles of bacteria causing bloodstream infection in neonates in Malawi between 1998 and 2017 revealed that resistance to standard first-line antibiotics (ampicillin/penicillin, gentamicin, ceftriaxone) changed from most organisms being sensitive, to most being resistant, with resistance rates in *Klebsiella* spp. exceeding 90%.^2^ Resistance to a third-generation cephalosporin such as cefotaxime is used to screen for an organism said to be producing ESBLs. Infection with MDR organisms including ESBL producers is associated with increased mortality in neonatal sepsis.^3^

The diagnosis of neonatal sepsis is challenging, with elusive and unsuitable definitions currently in use.^4^ Distinguishing between infectious and non-infectious causes, and in the infectious cases between viral and bacterial, relies heavily on microbial culture. Diagnostic microbial culture takes around 48 h, making it too slow to guide empirical treatment. Rapid diagnostics and combinations of biomarkers^5^ may partially reduce inappropriate empirical antibiotic use by identifying viral causes and allowing targeted treatment when organisms are known. In settings where resistance rates are high, there is an increasing trend to use very broad-spectrum agents such as carbapenems as first-line empirical treatment of neonatal sepsis^6^ but this approach risks driving further resistance acquisition. The WHO has categorized antimicrobials into ‘Access’, ‘Watch’ and ‘Reserve’ classes with the recommendation to limit the use of Watch and Reserve agents where possible.^7^

Of the currently used first-line agents for neonatal sepsis, gentamicin and ampicillin are in the Access group whereas cefotaxime is in the Watch group,^8^ and resistance to each of these agents individually has now reached alarming levels.^2^ However, standard antimicrobial susceptibility testing does not consider combinatorial effects. It is possible that the combination of two or more agents may have additive or synergistic activity sufficient to be effective against organisms that are resistant to each agent individually. Furthermore, adding a β-lactamase inhibitor (BLI) from the Access list such as sulbactam or clavulanic acid to a β-lactam antibiotic may also broaden the spectrum of activity sufficient to cover Gram-negative ESBL producers without resorting to carbapenems as empirical therapy.

This study therefore aimed to assess the activity of combinations of ampicillin, cefotaxime and gentamicin against *Escherichia coli* and *Klebsiella pneumoniae* clinical isolates known to have caused neonatal sepsis. In addition, the BLI sulbactam was tested due to it being commercially available in combination with either ampicillin or cefotaxime in a 2:1 ratio. If combination activity could be shown, it may indicate a possible empirical therapy for neonatal sepsis using mainly Access antibiotics.

## Materials and methods

### Materials

Thirty-four *E. coli* (designated EC01–34) and four *K. pneumoniae* (designated KC01–04) isolates were obtained from neonates suffering from sepsis in a large clinical trial^6^ and from hospitalized patients in the UK and Taiwan. All reagents, chemicals and antimicrobial agents were obtained from Sigma–Aldrich (Dorset, UK) unless otherwise stated. Mueller–Hinton broth (MHB) and tryptic soy broth (TSB) were used for general maintenance of *E. coli* and *K. pneumoniae* respectively, and Mueller–Hinton II broth (cation adjusted) (MHB2) was used in quantitative procedures for susceptibility testing for all isolates.

### Determination of MICs

Antimicrobial activity of commonly prescribed antibiotics was established against all isolates in accordance with the microbroth dilution method and disc diffusion method in accordance with EUCAST guidelines.^9,10^ MICs were determined of each of the following antibiotics/inhibitors: amikacin, ampicillin, cefotaxime, gentamicin, meropenem, sulbactam and clavulanic acid for each clinical isolate.

### Identification of resistance genes present in clinical isolates

The *E. coli* and *K. pneumoniae* clinical isolates found to be least susceptible to commonly administered antibiotics were selected and the presence or absence of commonly found resistance genes was established using PCR under standard conditions using previously published primers.^11,12^ Combined Illumina and Nanopore sequencing^13^ of *E. coli* (EC05, EC07, EC15, EC19, EC29, EC30, EC31 and EC32) and *K. pneumoniae* (KC01, KC02 and KC04) isolates was provided by MicrobesNG (http://www.microbesng.com), which is supported by the Biotechnology and Biological Sciences Research Council (BBSRC; grant number BB/L024209/1). These *E. coli and K. pneumoniae* isolates were selected for sequencing after being identified as having phenotypes of reduced susceptibility to commonly administered antibiotics. EC07 was found to be sensitive to gentamicin and not taken forward to the main panel.

Unicycler v0.4.0^14^ was used for hybrid *de novo* assembly of sequenced isolates. The resistance profile of assembled genomes was determined by screening against a ResFinder database.^15^

### Evaluation of synergy between two antibiotics

Evaluation of interactions between two-drug combinations against 15 *E. coli* and 3 *K. pneumoniae* clinical isolates was performed in a 96-well plate format using a standard 2D chequerboard layout.^16^ Plates were prepared with a final volume of 100 µL and a maximum concentration of each drug set at 2–4× MIC for each isolate, previously established by the microbroth dilution method. Doubling dilutions were performed along the abscissa (eight wells) and ordinate (eight wells) to provide concentration gradients. Each well was inoculated with a final bacterial cell density of 5 × 10^5^ cfu/mL and incubated at 37°C for 16–18 h. Bacterial cell density was estimated by reference to an OD_600_/cfu standard curve, and was verified by plating cell suspension dilutions onto Mueller-Hinton agar and cfu counted the next day after an overnight incubation at 37°C. Any starting inoculation that fell outside of the allowable 3–7 × 10^5^ cfu/mL range was rejected and the experiment repeated. If bacterial growth was present at well edges, the experiment was rejected and repeated using higher or lower drug concentrations where appropriate.

Bacterial growth or inhibition was determined by visual evaluation of turbidity^9^—any well that was more opaque than the media control was deemed to be subinhibitory. Positive (no drug) and negative (no bacteria) controls were included in the plate layout. Drug synergism was evaluated by calculation of the FIC index (FICI). The FICI is a standard method for assessing the interactions between two or more antimicrobial agents. It is used to determine whether the combination of antibiotics has a synergistic, indifferent or antagonistic effect on the growth of microorganisms.

FICI, with acknowledged limitations, has been widely used in microbiology research, and its use has been described in several publications.^17^ FICI is defined for two drugs as: MIC of drug 1 in combination with drug 2/MIC of drug 1 alone + MIC of drug 2 in combination with drug 1/MIC of drug 2 alone. Synergy was reported with an FICI value of lower than 0.5, no interaction or indifferent was defined as an FICI value of between 0.5 and 4, and an antagonistic interaction was defined as an FICI value of greater than 4.^18^

### Evaluation of synergy between three antibiotics

**Figure 1.**
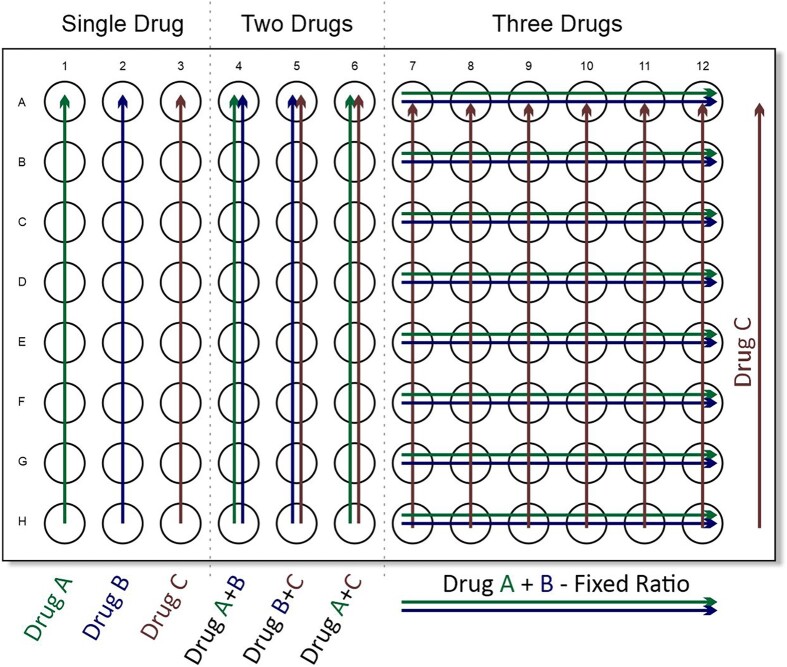
Ninety-six-well plate layout for synergy evaluation between three antimicrobial agents. Arrows represent increasing doubling drug concentration gradient. This figure appears in colour in the online version of *JAC* and in black and white in the print version of *JAC*.

A novel plate layout was designed to demonstrate additive, antagonistic or synergistic relationships between three antimicrobial combinations. One 96-well microtitre plate (VWR, Leicestershire, UK) was used to test all combinations of three selected antibiotics against a single *E. coli* or *K. pneumoniae* clinical isolate. An abbreviated 3D chequerboard layout (Figure 1) enabled evaluations of: (i) MICs for each individual drug; (ii) quantification of any synergistic relationships between each combination of pairs of drugs; and (iii) quantification of any synergy exhibited in a three-drug combination. Sulbactam was administered in a fixed ratio of 2:1 with cefotaxime or ampicillin to enable three-drug synergy evaluation without the need for multiple 96-well plates. Drug synergism was evaluated for pairs of two-drug combinations using the abbreviated 3D chequerboard plate and was calculated as described above in the 2D chequerboard format. For evaluation and quantification of possible synergy between three drugs, the FICI was calculated as: MIC of drug 1 in combination/MIC of drug 1 alone + MIC of drug 2 in combination/MIC of drug 2 alone + MIC of drug 3 in combination/MIC of drug 3 alone.^19^

### Hollow-fibre infection model conditions and methods

**Figure 2.**
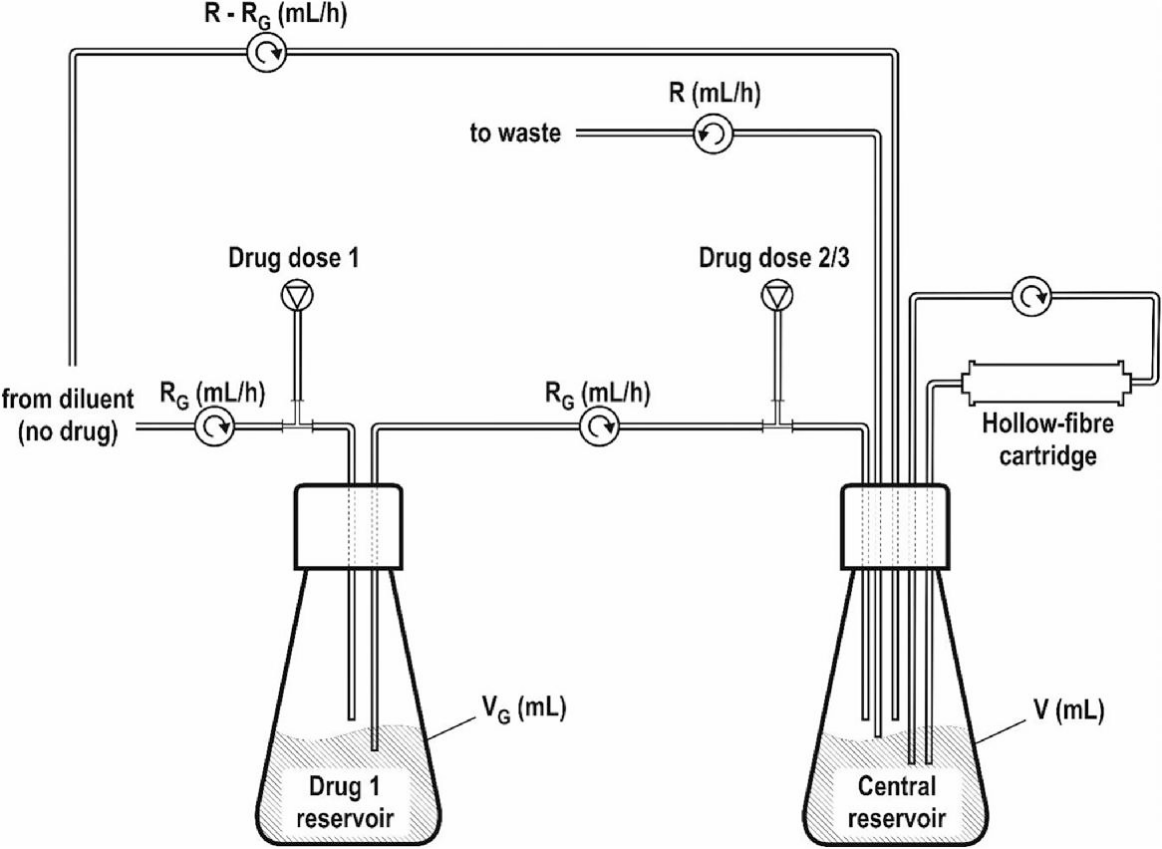
Schematic hollow-fibre system diagram incorporating an additional drug dosing reservoir. Hollow-fibre set-up for drugs with different half-lives adapted from Blaser^20^. R = main diluent flow rate (mL/h); R_G_ = flow rate for drug 1 (mg/L); V_G_ = volume of drug 1 reservoir (mL); V = volume of central reservoir (mL). To compensate for drug half-life differences in the hollow-fibre simulation system, the two-reservoir system was employed to enable the use of gentamicin in combination with ampicillin or cefotaxime under the following conditions: R = 12 mL/h; R_G_ = 6 mL/h; V_G_ = 100 mL; V = 100 mL.

A hollow-fibre system comprising a 20 kDa polysulfone fibre filtration module (Cellab GmbH, Radeberg, Germany; FiberCell Systems, MD, USA), central reservoir, diluent reservoir and an additional drug-dosing reservoir was used to evaluate bacterial antibiotic combination killing efficacy. Drugs were administered to the central and additional drug reservoir with automated syringe pumps (World Precision Instruments Ltd, Hertfordshire, UK); media circulation was controlled by a peristaltic or positive-displacement pump (Cole-Parmer Instrument Company Ltd, Cambridgeshire, UK; FiberCell Systems) with a circulation rate of approximately 25 mL/min. The dilution rate of the central reservoir varied depending upon the drugs used and clearance rate of the simulated target host organism and was controlled by an independent pump. The inclusion of a second independent media reservoir allowed the addition of an additional antibiotic with longer half-life than the drug(s) administered to the central reservoir (Figure 2) with rates calculated by methods adapted from Blaser.^20^

The dose administered to the central reservoir was set to obtain a typical preterm neonatal *C*_max_, which for cefotaxime was set to 180 mg/L,^21^ ampicillin 300 mg/L^22^ and 20 mg/L for gentamicin.^23^ When sulbactam was added to either cefotaxime or ampicillin, the *C*_max_ was set to one-third of that for cefotaxime or ampicillin. The half-lives of ampicillin, cefotaxime and sulbactam were assumed to be 6 h, whereas for gentamicin the half-life was assumed to be 12 h.^21,24^ The pump rates and volumes to achieve this are given in Figure 2. The central reservoir and cartridge circulation system was set to 100 mL total volume of MHB2. Bacterial inocula were prepared by overnight culture and dilution to yield 5 × 10^5^ cfu/mL in 20 mL total volume. The extracapillary space of the hollow-fibre cartridge was inoculated by an exchange of cartridge media for bacterial culture and the system incubated at 37°C along with all media reservoirs. In all experiments, bacterial cultures were allowed to reach a static growth phase (cell density approximately 1 × 10^10^ cfu/mL after 24 h) before being challenged with drug combinations.

Culture samples were aseptically taken over a typical experiment duration of 5–8 days. Timing and sample intervals were usually taken 1 h apart for the early logarithmic growth period, then fewer and as needed for the remainder of the duration of the experiment. Bacteria were quantified in each sample by culturing five 20 µL droplets of a range of dilutions onto Mueller–Hinton agar followed by overnight incubation at 37°C. The next day, dilutions containing at least 30 colonies per droplet were counted and cfu/mL calculated.

Experiments were monitored for bacterial or fungal contaminants by visual inspection of the central reservoir and diluent media; any contamination observed in the central reservoir or supplement reservoirs resulted in the termination of the experiment. Drug levels were quantified by LC/MS (Analytical Services International Ltd, London, UK) from media samples taken from the central reservoir at time intervals to represent a range of predicted high and low drug concentrations.

AUC_(0–7)_ of the log cfu/mL with time derived from the hollow fibre was calculated using the trapezoidal rule. Each drug regimen and control were compared by calculating the percentage change in AUC between control and drug-containing runs.^25^

## Results

### Antibiotic susceptibility profiles of panel of E. coli and K. pneumoniae clinical isolates

Susceptibility of the panel of 34 *E. coli* and 4 *K. pneumoniae* clinical isolates to amikacin, ampicillin, cefotaxime, gentamicin and meropenem was identified by the disc diffusion susceptibility test and MIC testing by microbroth dilution was used to quantify resistant isolates’ susceptibility to amikacin, ampicillin, cefotaxime, gentamicin, sulbactam and clavulanic acid (Table S1, available as Supplementary data at *JAC* Online). Seven *E. coli* and three *K. pneumoniae* isolates were found to be resistant to gentamicin, cefotaxime and ampicillin; the three *K. pneumoniae* isolates were additionally resistant to amikacin, and one was also resistant to meropenem (Table 1). The isolates with the least susceptible phenotypes were selected for further study.

**Table 1.** Summary of antimicrobial susceptibility of seven *E. coli* and three *K. pneumoniae* isolates

	MIC (microbroth dilution; mg/L)						Disc diffusion susceptibility test				
Isolate	AMK	AMP	CTX	GEN	SUL	CLA	AMK	AMP	CTX	GEN	MEM
*E. coli*											
EC05	**16**	**>2048**	**2048**	**256**	**64**	**128**	R	R	R	R	S
EC15	**16**	**>2048**	**64**	**128**	**64**	**128**	S	R	R	R	S
EC19	**8**	**>2048**	**>2048**	**256**	**64**	**128**	S	R	R	R	S
EC29	**16**	**2048**	**16**	**512**	**128**	**64**	S	R	R	R	S
EC30	**16**	**>2048**	**8**	**512**	**64**	**32**	S	R	R	R	S
EC31	**16**	**>2048**	**16**	**512**	**64**	**64**	S	R	R	R	S
EC32	**16**	**>2048**	**8**	**512**	**64**	**—**	R	R	R	R	S
*K. pneumoniae*											
KC01	**>1024**	**>1024**	**>2048**	**2048**	**128**	**—**	R	R	R	R	S
KC02	**128**	**>2048**	**128**	**8**	**256**	**—**	R	R	R	R	R
KC04	**>128**	**>2048**	**>2048**	**2048**	**64**	**—**	R	R	R	R	S

AMK, amikacin; AMP, ampicillin; CTX, cefotaxime; GEN, gentamicin; MEM, meropenem; SUL, sulbactam; CLA, clavulanic acid. Numbers represent antibiotic concentration (mg/L). Bold text in MIC columns indicates resistance, as defined by EUCAST (v13.0).^9^ Interpretation of sensitivity (S) or resistance (R) from disc zone diameters was in accordance with EUCAST clinical breakpoints (v13.0).^10^

### Identification of genes conferring reduced susceptibility to commonly used antibiotics

**Figure 3.**
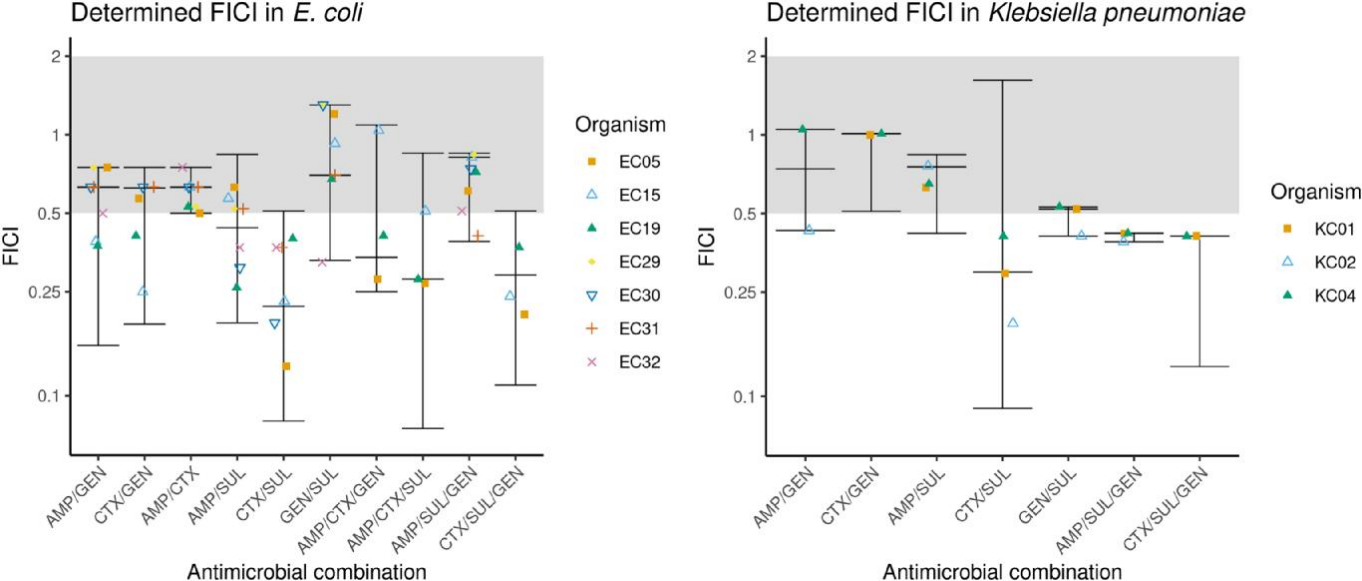
Median (middle bars) and range (top and bottom bars) of the FICI for *E. coli* (left) and *K. pneumoniae* (right). Shaded areas show the FICI range denoting additivity (0.5–2), whereas the white areas denote synergy (<0.5). This figure appears in colour in the online version of *JAC* and in black and white in the print version of *JAC*.

Genes encoding β-lactam and/or aminoglycoside resistance genes were detected by PCR (Table S2a) in isolates shown by MICs to have a profile of reduced susceptibility to commonly used antibiotics. Genes identified by WGS conferring reduced susceptibility to commonly used antibiotics are presented in Table S2b. The isolates with the least susceptible phenotypes contained a wide range of resistance genes, most notably β-lactamases (Table S2), and all 10 of the selected isolates were resistant to cefotaxime, ampicillin and gentamicin individually (Table 1). As observed in initial 2D chequerboard experiments, synergistic effects of two-drug combinations were often isolate-dependent and correlation with antibiotic resistance gene profiles was observed (Table S2 and Figure 3). Additionally, in some instances, even where a strong synergistic relationship was noted, drug concentrations required to inhibit growth were commonly too high to be regarded as equivalent to a viable therapeutic option. The effects of the combination of ampicillin and cefotaxime were often synergistic (FICI lower than 0.5) when used to inhibit the growth of isolates harbouring a single β-lactamase gene variant. They were consistently additive (FICI greater than 0.5 but lower than 4) against isolates expressing multiple β-lactamase variants (Figure 3). *E. coli* isolate EC07 was found by both PCR screening and WGS to harbour a single ESBL-encoding gene, *bla*_CTX-M-15_, and the combination of cefotaxime and ampicillin was observed to be atypically strongly synergistic (FICI = 0.13).

### Evaluation of synergy between antimicrobial agents against E. coli and K. pneumoniae

Evaluation of potential synergy between two drugs (two antibiotics, or one antibiotic plus sulbactam) was completed using a standard 2D chequerboard layout for two-drug combinations in a 96-well plate format. Synergy between combinations of two agents acted synergistically in 24 out of 60 (Figure 3) drug pairs, but the effect was isolate-dependent and there were no combinations that were universally synergistic across the selected panel of *E. coli* and *K. pneumoniae* clinical isolates.

To potentially reduce inhibitory concentrations of individual drugs to the equivalent of a clinically usable dose, combinations of three drugs were investigated. An abbreviated 3D 96-well plate layout for three-drug combinations (Figure 1) was used to establish synergistic, indifferent, or antagonistic relationships between three agents. As noted with combinations of two drugs, synergistic relationships were isolate-dependent with some combinations consistently synergistic in one isolate and additive in another. Interestingly, the addition of a third drug reduced the concentration of individual antimicrobial agents to levels that potentially fell into the equivalent of a clinically usable range. The combination of cefotaxime/sulbactam/gentamicin (CTX/SUL/GEN) was the most consistently synergistic when tested against most *E. coli* and *K. pneumoniae* isolates (16/17 isolates). Evaluation of the combination of ampicillin/sulbactam/gentamicin (AMP/SUL/GEN) indicated an additive relationship in most experiments (7/8 *E. coli* experiments; Figure 3). In experiments where synergy was observed, concentrations of ampicillin required to inhibit growth were often high (56.25 mg/L in all three *K. pneumoniae* experiments). In contrast, the combination of CTX/SUL/GEN was the only combination in which synergy most often observed, and inhibitory drug concentrations were reduced into a clinically usable range.

### Effects on bacterial growth of three-drug combinations in a hollow-fibre infection model

**Figure 4.**
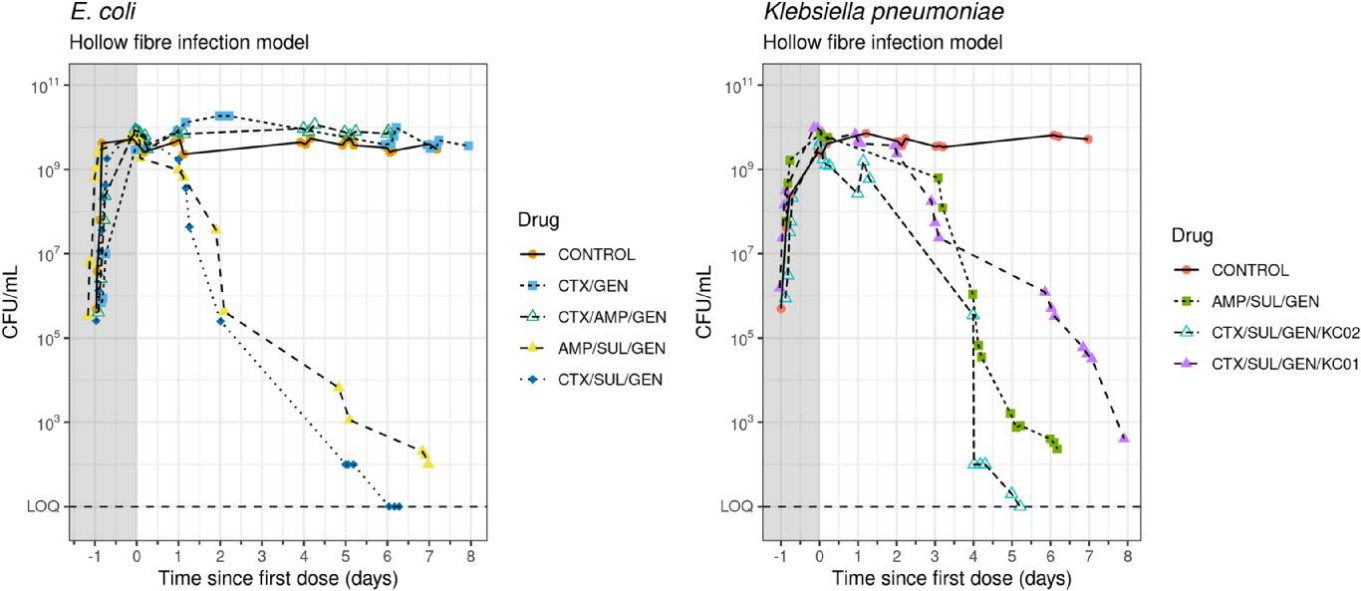
Effects of combinations of antibiotics on *E. coli* clinical isolate EC19 in a hollow-fibre infection model (left) and on *K. pneumoniae* clinical isolates (KC01 and KC02) in a hollow-fibre infection model. The shaded areas represent the 24 h pre-dose period. Different symbols and line types represent different antimicrobial combinations. This figure appears in colour in the online version of *JAC* and in black and white in the print version of *JAC*.

For evaluation of two- and three-drug combinations against *E. coli* in a hollow-fibre infection model, isolate EC19 was selected as the clinical isolate with the phenotype of lowest susceptibility to amikacin, ampicillin, cefotaxime and gentamicin (Table 1), as established by MIC and synergy testing (Table 1; Figure 3). The inoculated hollow-fibre system was incubated at 37°C for 24 h before the administration of the drug combination. No bactericidal effects were observed in comparison with the growth control with the combinations of cefotaxime/gentamicin and cefotaxime/ampicillin/gentamicin (CTX/AMP/GEN) after 7 days, but bactericidal effects (greater than a 3 log^10^ decline in population density) were observed with cultures challenged with either AMP/SUL/GEN or CTX/SUL/GEN combinations (Figure 4). The sulbactam-containing regimens yielded approximately 36% and 48% decline in cfu AUC for AMP/SUL/GEN and CTX/SUL/GEN, respectively, in *E. coli*.

Hollow-fibre experiments were undertaken to investigate the bactericidal effects of two-drug plus sulbactam combinations on *K. pneumoniae* clinical isolates KC01 and KC02. The combination of CTX/SUL/GEN was bactericidal against KC01 and KC02 bacterial populations. The combination of AMP/SUL/GEN (300/150/20 mg/L) was also bactericidal against *K. pneumoniae* clinical isolate KC01 culture. The growth control (no drugs) showed no decline in population density over the experiment duration (Figure 4). cfu AUC decreases compared with control were 37% and 51% for AMP/SUL/GEN and CTX/SUL/GEN in KC02 and 22% for KC01 treated with CTX/SUL/GEN.

By the seventh day, bacterial clearance (no detection of bacterial growth above the detection limit) was complete with CTX/SUL/GEN for both *E. coli* and *K. pneumoniae*; no sustained killing effect was seen with cefotaxime plus gentamicin. The triple combination of CTX/AMP/GEN did not produce sustained killing despite showing chequerboard synergy, indicating the need for sulbactam. The addition of sulbactam to two-drug combinations, administered in a 2:1 ratio with either cefotaxime or ampicillin, decreased the concentrations of each component drug required to exhibit bactericidal effects. The concentration of cefotaxime/sulbactam in the CTX/SUL/GEN combination required to achieve bacterial clearance was consistently lower, and fell in the range equivalent to a clinically achievable concentration, than that of ampicillin/sulbactam in the AMP/SUL/GEN combination required to achieve a comparable bactericidal effect. Concentrations of ampicillin/sulbactam (300/150 mg/L) in the AMP/SUL/GEN combination in hollow-fibre experiments were necessarily higher than those of cefotaxime/sulbactam (180/90 mg/L) in the CTX/SUL/GEN combination. With lower concentrations, the CTX/SUL/GEN combination achieved a bacterial culture decline to or below the limit of detection in 5–6 days, whereas the AMP/SUL/GEN combination was insufficient to achieve the same level of clearance in 6–7 days (Figure 4).

The dosing concentrations of CTX/SUL/GEN selected as being equivalent to clinically achievable levels was consistently sufficient to achieve bactericidal effects (greater than a 3 log^10^ decline in population density) in hollow-fibre experiments.

## Discussion

Antimicrobial resistance is a significant and increasing problem in neonatal settings. There is an urgent need for improved treatments. Combination antibiotic therapy has not yet been extensively studied. We have shown that cefotaxime/sulbactam or ampicillin/sulbactam combined with gentamicin is a potentially useful empirical first-line regimen for use in neonatal sepsis where ESBL prevalence is high. Whilst previous studies have looked at individual drug resistance and concluded that combinations of agents are not useful if an organism is resistant to each drug individually,^2^ we have shown that additivity and synergy within CTX/SUL/GEN combinations at usual neonatal doses can sterilize the hollow-fibre infection model within 7 days despite isolates being individually resistant to each drug. AMP/SUL/GEN combinations were also bactericidal, but the rate of bacterial clearance was slower than that of CTX/SUL/GEN.

Chequerboard and hollow-fibre results showed cefotaxime/sulbactam with gentamicin was consistently bactericidal at clinically achievable concentrations across the panel of *E. coli* and *K. pneumoniae* isolates tested. This effect was observed at bacterial concentrations greater than would usually be expected in a neonatal infection.

Observed inhibitory concentrations of individual drugs, i.e. cefotaxime and ampicillin, in a two-drug combination with sulbactam, were often too high to be regarded as clinically achievable, even where strong synergy was indicated. For example, in 2D chequerboard experiments with isolate EC30, cefotaxime/sulbactam and ampicillin/sulbactam FICI were both 0.19 (synergistic). The concentrations required to inhibit growth of the ampicillin/sulbactam combination were higher than clinically usable ( 562.5 mg/L ampicillin and 3.52 mg/L sulbactam).

In contrast, the same isolate with the same FICI of 0.19 required concentrations of 6.25 mg/L cefotaxime and 4.69 mg/L sulbactam to achieve the same inhibitory effect. The addition of gentamicin reduced component drug concentrations into a clinically usable range.

To study triple combinations, the FICI was extended and required the development of a novel plate layout to ensure a sufficient range of drug concentrations and combinations could be studied (Figure 1). The combination of an agent that reduces bacterial protein production through ribosome binding (gentamicin) and a β-lactam antibiotic was generally additive with a trend towards synergy (Figure 3), but this was not sufficient to inhibit growth of the most resistant *E. coli* (EC19) at clinical concentrations in the hollow-fibre model (Figure 4).

Observed inhibitory concentrations of individual drugs, i.e. cefotaxime and ampicillin, in a two-drug combination with sulbactam, were often too high to be regarded as clinically achievable, even where strong synergy was indicated. For example, in two E. coli isolates, 2D chequerboard experiments revealed synergy (FICI = 0.9 and 0.12) with the minimum required concentrations of the cefotaxime contribution to the combination being 128 mg/L in both cases. The addition of gentamicin reduced overall component drug concentrations into a clinically usable range.

Greater cross-isolate consistency was observed with three-drug combinations but the double β-lactam combination of cefotaxime/ampicillin with the addition of gentamicin was unable to sterilize the hollow-fibre cartridge with *E. coli* isolate EC19. The minimum observed FICI for this combination was 0.25. The most common mechanism of resistance to antibiotics is the expression of β-lactamases, which bind to and degrade β-lactam antibiotics.^26^ Sulbactam is a synthetic β-lactamase inhibitor that contains a penicillin-like β-lactam ring structure. Whilst demonstrating low antimicrobial activity when used alone, sulbactam shows direct activity against *Bacteroides* and *Acinetobacter* species.^27^ It is an irreversible inhibitor of a variety of β-lactamases and has proved successful in broadening the spectrum of activity of certain antibiotics and reversing β-lactamase-mediated resistance.^28^

The high level of synergy observed with CTX/AMP/GEN against EC19, harbouring the ESBL-producing gene *bla*_CTX-M-15_ in addition to broad-spectrum β-lactamases *bla*_TEM-1B_ and *bla*_OXA-1_, may potentially be caused by the high activity of *bla*_CTX-M-15_ against cefotaxime.^29^ In this environment, cefotaxime may be primarily performing the function of a BLI, and effectively increasing the activity of ampicillin, sulbactam and gentamicin. It would be not unexpected to observe that in some specific phenotypic environments, other β-lactam antibiotic combinations are more synergistic and exhibit greater bactericidal efficacy than a β-lactam/sulbactam combination; however, sulbactam inhibits a variety of β-lactamases and synergy with other β-lactam antibiotics is consistently observed in a broad spectrum of isolates and species.

Unlike previous hollow-fibre reports, we chose to allow bacteria to grow for 24 h before adding antimicrobials. The bacterial densities thus encountered were significantly higher than would be encountered *in vivo* (10^10^ versus 10^3^)^30^ and the fact that the cartridge could be sterilized with such a high initial inoculum shows how potent the CTX/SUL/GEN combination is. By allowing the organisms to reach stationary phase, we used a very high initial inoculum in the hollow-fibre infection model. Thus, our experiments were not able to detect bacteriostatic effects. Having said this, bactericidal activity may be preferable in neonates with developing immune systems, but future experiments with CTX/SUL/GEN on other strains should consider starting therapy during log-phase growth.

The main limitation of our work was the low number of isolates tested, albeit these were all organisms isolated from neonates with sepsis. We chose isolates that were resistant to each drug individually for combination testing as ones that were sensitive to any one component of the combination would have been growth inhibited. From the available clinical isolates we chose the most strongly resistant to cefotaxime, ampicillin and gentamicin. Clinical isolates EC19, KC01 and KC02 had different genotypes harbouring varieties of β-lactamase and aminoglycoside resistance genes (Table S2).

Further experiments using a wider range of ESBL-producing isolates may be useful, and clinical trials are warranted and further investigations should be undertaken with a greater range of bacterial organisms and antibiotic resistance profiles. Whilst we did not undertake a separate pharmacokinetic sampling run, we did sample the central reservoir from one of the bacterial runs (Figure S1), showing that the pump settings seemed to correctly deliver initial doses, but lower levels of cefotaxime and sulbactam at later timepoints possibly indicate β-lactamase degradation. A limitation of our work is that we did not perform detailed pharmacokinetic sampling, but our quality assurance procedures ensuring doses and pump settings were rigorously checked and there were regular checks for leaks, along with the fact that lower later concentrations indicated the system was, if anything, under- rather than over-dosed, likely means our results are robust. Future work understanding the dynamics of β-lactamase induction with this regimen is warranted. A major next step in implementing CTX/SUL/GEN in a clinical setting is to develop a diagnostic test, since we have shown that organisms which are resistant/resistant/resistant are potentially treatable. The first step would be to gather sufficient data to set a breakpoint for cefotaxime/sulbactam, and then develop a test that also incorporates gentamicin. Such a test at present might include a custom disc-diffusion assay with discs containing CTX/SUL/GEN.

### Conclusions

The addition of sulbactam to the standard ampicillin/gentamicin or cefotaxime/gentamicin regimen at standard doses is a promising combination for neonatal settings with high ESBL prevalence where sulbactam is not already in use. Eradication of viable bacteria within 7 days was seen with standard doses, indicating that the commercially available ratio of ampicillin/sulbactam and cefotaxime/sulbactam (2:1) is likely to be adequate. These results suggest that the combination of CTX/SUL/GEN could represent an effective strategy against sepsis-causing Enterobacteriaceae infection at clinically achievable concentrations. The administration of combinations of CTX/SUL/GEN to combat MDR bacterial infections may be a potential alternative to using Watch agents such as amikacin and meropenem.

## Supplementary Material

dkad177_Supplementary_Data

## Supplementary data

Figure S1 and Tables S1 and S2 are available as Supplementary data at *JAC* Online.

## References

[1] Droz N, Hsia Y, Ellis S et al Bacterial pathogens and resistance causing community acquired paediatric bloodstream infections in low- and middle-income countries: a systematic review and meta-analysis. Antimicrob Resist Infect Control 2019; 8: 207. 10.1186/s13756-019-0673-5 31893041 PMC6937962

[2] Tam PYI, Musicha P, Kawaza K et al Emerging resistance to empiric antimicrobial regimens for pediatric bloodstream infections in Malawi (1998–2017). Clin Infect Dis 2019; 69: 61–8. 10.1093/cid/ciy834 30277505 PMC6579959

[3] Investigators of the Delhi Neonatal Infection Study (DeNIS) collaboration . Characterisation and antimicrobial resistance of sepsis pathogens in neonates born in tertiary care centres in Delhi, India: a cohort study. Lancet Global Health 2016; 4: e752–60. 10.1016/S2214-109X(16)30148-6 27633433

[4] McGovern M, Giannoni E, Kuester H et al Challenges in developing a consensus definition of neonatal sepsis. Pediatr Res 2020; 88: 14–26. 10.1038/s41390-020-0785-x 32126571

[5] Ruan L, Chen GY, Liu Z et al The combination of procalcitonin and C-reactive protein or presepsin alone improves the accuracy of diagnosis of neonatal sepsis: a meta-analysis and systematic review. Crit Care 2018; 22: 316. 10.1186/s13054-018-2236-1 30463590 PMC6249912

[6] Lutsar I, Chazallon C, Trafojer U et al Meropenem vs standard of care for treatment of neonatal late onset sepsis (NeoMero1): a randomised controlled trial. PLoS One 2020; 15: e0229380. 10.1371/journal.pone.0229380 PMC7055900 32130261

[7] Sharland M, Pulcini C, Harbarth S et al Classifying antibiotics in the WHO essential medicines list for optimal use—be AWaRe. Lancet Infect Dis 2018; 18: 18–20. 10.1016/S1473-3099(17)30724-7 29303731

[8] WHO . WHO releases the 2019 AWaRe classification antibiotics. 2019. https://www.who.int/news/item/01-10-2019-who-releases-the-2019-aware-classification-antibiotics.

[9] EUCAST . EUCAST broth microdilution reading guide ,version 4.0 (January 2022). http://www.eucast.org.

[10] EUCAST . EUCAST disk diffusion method for antimicrobial susceptibility testing, version 11.0 (January 2023). http://www.eucast.org. 10.1111/apm.70002 PMC11807598 39923774

[11] Dallenne C, Da Costa A, Decré D et al Development of a set of multiplex PCR assays for the detection of genes encoding important β-lactamases in Enterobacteriaceae. J Antimicrob Chemother 2010; 65: 490–5. 10.1093/jac/dkp498 20071363

[12] Mir AR, Bashir Y, Dar FA et al Identification of genes coding aminoglycoside modifying enzymes in *E. coli* of UTI patients in India. Scientific World Journal 2016; 2016: 1875865. 10.1155/2016/1875865 PMC4926017 27403451

[13] MicrobesNG. MicrobesNG—Genome Sequencing Service Methods. 2021. https://microbesng.com/documents/24/MicrobesNG_Sequencing_Service_Methods_v20210419.pdf.

[14] Wick RR, Judd LM, Gorrie CL et al Unicycler: resolving bacterial genome assemblies from short and long sequencing reads. PLoS Comput Biol 2017; 13:e1005595. 10.1371/journal.pcbi.1005595 PMC5481147 28594827

[15] Bortolaia V, Kaas RS, Ruppe E et al ResFinder 4.0 for predictions of phenotypes from genotypes. J Antimicrob Chemother 2020; 75: 3491–500. 10.1093/jac/dkaa345 32780112 PMC7662176

[16] Bonapace CR, Bosso JA, Friedrich LV et al Comparison of methods of interpretation of checkerboard synergy testing. Diagn Microbiol Infect Dis 2002; 44: 363–6. 10.1016/S0732-8893(02)00473-X 12543542

[17] Odds FC . Synergy, antagonism, and what the chequerboard puts between them. J Antimicrob Chemother 2003; 52: 1. 10.1093/jac/dkg301 12805255

[18] Gómara M, Ramón-García S. The FICI paradigm: correcting flaws in antimicrobial *in vitro* synergy screens at their inception. Biochem Pharmacol 2019; 163: 299–307. 10.1016/j.bcp.2019.03.001 30836058

[19] Stein C, Makarewicz O, Bohnert JA et al Three dimensional checkerboard synergy analysis of colistin, meropenem, tigecycline against multidrug-resistant clinical *Klebsiella pneumonia* isolates. PLoS One 2015; 11: e0126479. 10.1371/journal.pone.0126479 PMC4465894 26067824

[20] Blaser J . *In-vitro* model for simultaneous simulation of the serum kinetics of two drugs with different half-lives. J Antimicrob Chemother 1985; 15: 125–30. 10.1093/jac/15.suppl_A.125 3980323

[21] Pacifici G, Marchini G. Clinical pharmacology of cefotaxime in neonates and infants: effects and pharmacokinetics. Int J Pediatr 2017; 5: 6111–38. 10.22038/ijp.2017.26241.2244

[22] Tremoulet A, Le J, Poindexter B et al Characterization of the population pharmacokinetics of ampicillin in neonates using an opportunistic study design. Antimicrob Agents Chemother 2014; 58: 3013–20. 10.1128/AAC.02374-13 24614374 PMC4068432

[23] Germovsek E, Kent A, Metsvaht T et al Development and evaluation of a gentamicin pharmacokinetic model that facilitates opportunistic gentamicin therapeutic drug monitoring in neonates and infants. Antimicrob Agents Chemother 2016; 60: 4869–77. 10.1128/AAC.00577-16 27270281 PMC4958175

[24] Pacifici GM, Marchini G. Clinical pharmacokinetics of gentamicin in neonates. Int J Pediatr 2017; 5: 4575–99.

[25] MacGowan AP, Noel AR, Rogers CA et al Antibacterial effects of amoxicillin-clavulanate against *Streptococcus pneumoniae* and *Haemophilus influenzae* strains for which MICs are high, in an in vitro pharmacokinetic model. Antimicrob Agents Chemother 2004; 48: 2599–603. 10.1128/AAC.48.7.2599-2603.2004 15215115 PMC434219

[26] Sandanayaka VP, Prashad AS. Resistance to β-lactam antibiotics: structure and mechanism based design of β-lactamase inhibitors. Curr Med Chem 2002; 9: 1145–65. 10.2174/0929867023370031 12052169

[27] Williams JD . β-Lactamase inhibition and in vitro activity of sulbactam and sulbactam/cefoperazone. Clin Infect Dis 1997; 24: 494–7. 10.1093/clinids/24.3.494 9114205

[28] Drawz SM, Bonomo RA. Three decades of β-lactamase inhibitors. Clin Microbiol Rev 2010; 23: 160–201. 10.1128/CMR.00037-09 20065329 PMC2806661

[29] Bauernfeind A, Holley M, Jungwirth R et al A new plasmidic cefotaximase from patients infected with *Salmonella typhimurium*. Infection 1992; 20: 158–63. 10.1007/BF01704610 1644493

[30] Dietzman DE, Fischer GW, Schoenknecht FD. Neonatal *Escherichia coli* septicemia–bacterial counts in blood. J Pediatr 1974; 85: 128–30. 10.1016/S0022-3476(74)80308-2 4604810

