# Supplementary material for: Cefotaxime/sulbactam plus gentamicin as a potential carbapenem- and amikacin-sparing first-line combination for neonatal sepsis in high ESBL prevalence settings
Source: J Antimicrob Chemother. 2023 Jun 7;78(8):1882–90. doi: 10.1093/jac/dkad177 (PMC10393883; doi:10.1093/jac/dkad177)
Supplement: dkad177_Supplementary_Data [file dkad177_supplementary_data.docx]

**Supplementary data**

Table S1. Summary of antimicrobial susceptibility of *E. coli* and *K. pneumoniae* isolates to AMK, AMP, CTX, GEN, MEM, SUL and CLA. Numbers represent antibiotic concentration (mg/L). Interpretation of sensitivity (S) or resistance (R) from disk zone diameters were in accordance with EUCAST clinical breakpoints (v13.0).

|  | **MIC (Micro-broth dilution; mg/L)** | | | | | | **Disk Diffusion Susceptibility Test** | | | | |  |
| --- | --- | --- | --- | --- | --- | --- | --- | --- | --- | --- | --- | --- |
| **Isolate** | **AMK** | **AMP** | **CTX** | **GEN** | **SUL** | **CLA** | **AMK** | **AMP** | **CTX** | **GEN** | **MEM** |  |
| ***Escherichia coli*** | | | | | | | | | | | |  |
| EC01 | 4 | 4096 | <0.125 | 8 |  | 128 | S | R | S | S |  |  |
| EC02 | 8 | 2 | 0.125 | 4 |  | 128 | S | S | S | S |  |  |
| EC03 | 8 | 128 | 4 | 2 |  | 64 | S | R | S | S |  |  |
| EC04 | 8 | 32 | 0.125 | 8 |  | 128 | R | S | S | R |  |  |
| EC05 | 16 | >2048 | 2048 | 128 | 64 | 128 | R | R | R | R | S |  |
| EC06 | 8 | 8 | 1 | 8 |  | 64 | S | S | S | R |  |  |
| EC07 | 32 | >2048 | 4096 | 4 |  | 64 | R | R | R | S |  |  |
| EC08 | 4 | 8 | 64 | 4 |  | 128 | S | S | S | S |  |  |
| EC09 |  | 16 | <0.25 | 1 |  |  | S | R | S | S | S |  |
| EC10 |  | >2048 |  | 4 |  | 64 | S | R | S | S | S |  |
| EC11 | 4 | >2048 | 8 | 0.125 |  | 64 | S | R | S | S | S |  |
| EC12 |  | >2048 | 4 | 16 |  | 128 | R | R | S | R | S |  |
| EC13 |  | >2048 | 1 |  | 64 | 64 | S | R | S | R | S |  |
| EC14 |  |  |  |  |  | 64 | S | S | S | S | S |  |
| EC15 | >1 | >2048 | 64 | 128 | 128 | 128 | S | R | R | R | S |  |
| EC16 |  | >2048 | 8 | 4 |  | 64 | S | R | S | S | S |  |
| EC17 |  | >2048 |  | 4 |  | 128 | S | R | S | S | S |  |
| EC18 |  | >2048 | 4 |  |  | 4 | S | R | S | S | S |  |
| EC19 | 64 | >2048 | >2048 | >2048 |  | 128 | S | R | R | R | S |  |
| EC20 |  | >2048 | 8 | 2 |  | 2 | S | R | S | S | S |  |
| EC21 |  | >2048 | 1 | 256 |  | 4 | S | R | S | R | S |  |
| EC22 |  | >2048 | 64 | 16 | 512 | 16 | S | R | R | R | S |  |
| EC23 |  | >2048 |  | 4 |  | 4 | S | R | S | S | S |  |
| EC24 |  | 2048 |  | 256 |  | 2 | R | R | S | R | S |  |
| EC25 |  |  |  |  |  |  | S | S | S | S | S |  |
| EC26 |  | 8 |  | 8 |  |  | S | R | S | S | S |  |
| EC27 |  |  |  |  |  | 4 | S | S | S | S | S |  |
| EC28 |  | 8 |  | 8 |  | 2 | S | R | S | S | S |  |
| EC29 | 16 | >2048 | 16 | 512 |  | 64 | S | R | R | R | S |  |
| EC30 | 16 | >2048 | 8 | 1024 | 64 | 32 | S | R | R | R | S |  |
| EC31 | 16 | >2048 | 16 | 512 | 64 | 64 | S | R | R | R | S |  |
| EC32 | 16 | >2048 | 8 | 1024 | 64 |  | R | R | R | R | S |  |
| EC33 |  | >128 | 1 | 1 |  |  | S | S | S | S | S |  |
| EC34 |  |  |  |  |  | 2 | S | S | S |  | S |  |

| ***Klebsiella pneumoniae*** | | | | | | | | | | | |
| --- | --- | --- | --- | --- | --- | --- | --- | --- | --- | --- | --- |
| KC01 | >1024 | >1024 | >2048 | >2048 | 128 |  | R | R | R | R | S |
| KC02 | 128 | >2048 | 256 | 8 | 256 |  | R | R | R | R | R |
| KC03 | 4 |  | 16 | 4 | 256 |  | S | R | S | R | S |
| KC04 | >128 | >2048 | >2048 | >2048 | 128 |  | R | R | R | R | S |

Table S2. A) Resistance genes identified in clinical isolates by PCR. Genes/ gene groups positively identified as present are indicated by a ‘+’. Genes/groups not identified are indicated by ‘-‘. No symbol indicates no PCR was completed for that isolate/gene combination. B) Resistance genes identified in clinical isolates by whole genome sequencing. Genes listed in column one are indicated as present by a ‘+’ in the column of each isolate.

|  |  | | EC05 | EC07 | EC15 | EC19 | EC29 | EC30 | EC31 | EC32 | KC01 | KC02 | KC04 |
| --- | --- | --- | --- | --- | --- | --- | --- | --- | --- | --- | --- | --- | --- |
| **A)** | TEM variants including TEM-1 and TEM-2 | **-** | | **-** | **-** | **+** | **+** | **+** | **+** | **+** | **-** | **-** | **+** |
|  | SHV variants including SHV-1 | **-** | | **-** | **-** | **-** | **-** | **-** | **-** | **-** | **-** | **+** | **+** |
|  | OXA-1, OXA-4, OXA-30 | **+** | | **-** | **-** | **+** | **-** | **-** | **-** | **-** | **-** | **+** | **-** |
|  | Variants of CTX-M group 1 including 1, 3 & 15 | **+** | | **+** | **-** | **+** | **-** | **-** | **-** | **-** | **+** | **-** | **+** |
|  | Variants of CTX-M group 2 | **-** | | **-** | **-** | **-** | **-** | **-** | **-** | **-** | **-** | **-** | **-** |
|  | Variants of CTX-M group 9 including 9 & 14 | **-** | | **-** | **-** | **-** | **-** | **-** | **-** | **-** | **-** | **-** | **-** |
|  | CTX-M-group 8/25 | **-** | | **-** | **-** | **-** | **-** | **-** | **-** | **-** | **-** |  |  |
|  | aacC1 | **-** | | **-** | **-** | **-** | **-** | **-** | **-** | **-** |  |  |  |
|  | aacC2 | **+** | | **+** | **+** | **+** | **+** | **+** | **+** | **+** | **-** | **-** | **-** |
|  | aacC3 | **-** | | **-** | **-** | **-** | **-** | **-** | **-** | **-** | **-** | **-** | **-** |
|  | aacC4 | **-** | | **-** | **-** | **-** | **-** | **-** | **-** | **-** | **-** | **-** | **-** |
|  | aadC | **-** | | **-** | **-** | **-** | **-** | **-** | **-** | **-** | **-** | **-** | **-** |
|  | aacA - aphD | **-** | | **-** | **-** | **-** | **-** | **-** | **-** | **-** |  |  |  |
|  | aphA3 | **-** | | **-** | **-** | **-** | **-** | **-** | **-** | **-** |  |  |  |
| **B)** | aac(3)-IIa | **+** | |  | **+** | **+** |  |  |  |  |  |  |  |
|  | aac(3)-IId |  | |  |  |  | **+** | **+** | **+** | **+** |  |  |  |
|  | aac(6')-Ib-cr | **+** | |  | **+** | **+** |  |  |  |  |  |  |  |
|  | aadA5 | **+** | |  | **+** | **+** |  |  |  |  |  |  |  |
|  | ARR-3 |  | |  |  |  |  |  |  |  |  | **+** |  |
|  | blaCMY-2 |  | |  |  |  | **+** | **+** | **+** | **+** |  |  |  |
|  | blaCTX-M-15 | **+** | | **+** | **+** | **+** |  |  |  |  | **+** | **+** | **+** |
|  | blaOXA-1 | **+** | |  | **+** | **+** |  |  |  |  |  | **+** |  |
|  | blaSHV-28 |  | |  |  |  |  |  |  |  | **+** |  | **+** |
|  | blaSHV-94 |  | |  |  |  |  |  |  |  |  | **+** |  |
|  | blaTEM-1B |  | |  | **+** | **+** | **+** | **+** | **+** | **+** | **+** |  | **+** |
|  | catA1 |  | |  | **+** | **+** |  |  |  |  | **+** |  | **+** |
|  | catB3 |  | |  |  |  |  |  |  |  |  | **+** |  |
|  | dfrA17 | **+** | |  | **+** | **+** |  |  |  |  |  |  |  |
|  | fosA |  | |  |  |  |  |  |  |  | **+** | **+** | **+** |
|  | mdf(A) | **+** | | **+** | **+** | **+** | **+** | **+** | **+** | **+** |  |  |  |
|  | mph(A) | **+** | |  | **+** | **+** |  |  |  |  |  |  |  |
|  | oqxA |  | |  |  |  |  |  |  |  | **+** | **+** | **+** |
|  | oqxB |  | |  |  |  |  |  |  |  | **+** | **+** | **+** |
|  | qepA1 |  | |  |  |  |  |  |  |  |  |  | **+** |
|  | qnrE1 |  | |  |  |  |  |  |  |  | **+** |  | **+** |
|  | qnrS1 |  | | **+** |  |  |  |  |  |  |  |  |  |
|  | rmtB |  | |  |  |  |  |  |  |  | **+** |  | **+** |
|  | sul1 | **+** | |  | **+** | **+** |  |  |  |  |  | **+** |  |
|  | tet(B) |  | |  | **+** | **+** |  |  |  |  |  | **+** |  |


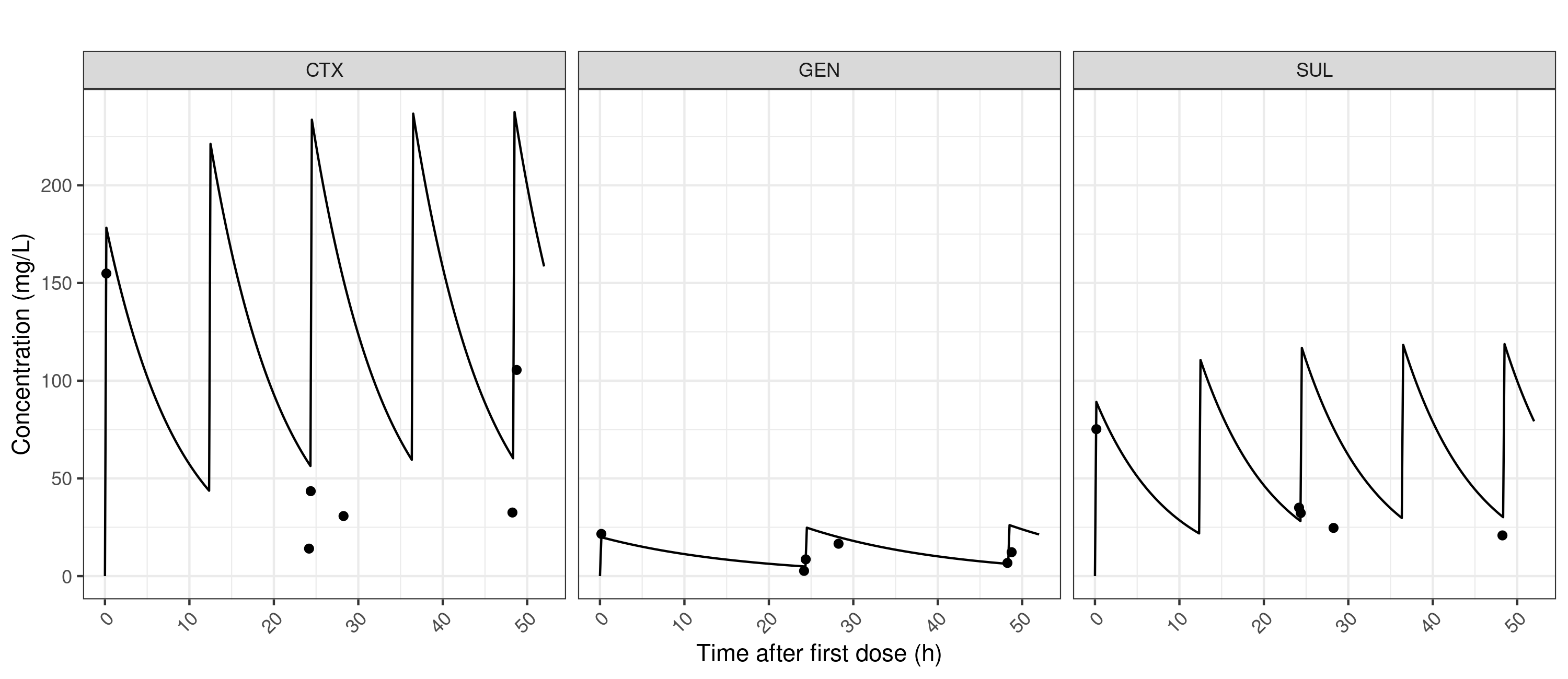
 Figure S1: Pharmacokinetic samples taken during EC19 treated with CTX/SUL/GEN from the central reservoir measured by liquid chromatography/mass spectroscopy. Lines are model predictions based on pump settings, black circles are measured concentrations. Whilst GEN and initial CTX and SUL concentrations are predicted well, later CTX and SUL concentrations are lower than expected despite this combination going on to sterilise the cartridge, possibly indicating degradation of β-lactams by β-lactamases inducted by treatment.
